# Investigating dopaminergic abnormalities in schizophrenia and first-episode psychosis with normative modelling and multisite molecular neuroimaging

**DOI:** 10.1038/s41380-025-02938-w

**Published:** 2025-02-28

**Authors:** Alessio Giacomel, Daniel Martins, Giovanna Nordio, Rubaida Easmin, Oliver Howes, Pierluigi Selvaggi, Steven C. R. Williams, Federico Turkheimer, Marius De Groot, Ottavia Dipasquale, Mattia Veronese, the FDOPA PET imaging working group, Ilinca Angelescu, Micheal Bloomfield, Ilaria Bonoldi, Faith Borgan, Tarik Dahoun, Enrico D’Ambrosio, Arsime Demjaha, Jecek Donocik, Alice Egerton, Sameer Jauha, Stephen Kaar, Euitae Kim, Seoyoung Kim, James Maccabe, Julian Matthews, Robert McCutcheon, Philip McGuire, Chiara Nosarti, Matthew Nour, Maria Rogdaki, Grazia Rutigliano, Peter S. Talbot, Luke Vano

**Affiliations:** 1 Centre for Neuroimaging Sciences, Institute of Psychology, Psychiatry and Neuroscience (IoPPN), King’s College London, London, UK; 2 Division of Adult Psychiatry, Department of Psychiatry, Geneva University Hospitals, Rue Gabrielle Perret-Gentil 4, 1205 Geneva, Switzerland; 3 Department of Psychosis Studies, Institute of Psychology, Psychiatry and Neuroscience (IoPPN), King’s College London, London, UK; 4 MRC Laboratory of Medical Science, Imperial College London, London, UK; 5 South London and Maudsley NHS Foundation Trust, London, UK; 6 Department of Translational Biomedicine and Neuroscience, University of Bari “Aldo Moro”, Bari, Italy; 7 GSK R&D, Clinical Pharmacology and Experimental Medicine, Clinical Imaging, Stevenage, UK; 8 Department of Information Engineering, University of Padova, Padova, Italy; 9 Department of Psychosis Studies, Institute of Psychiatry, Psychology & Neuroscience, King’s College London, London, UK; 10 Division of Psychiatry, Faculty of Brain Sciences, University College of London, London, UK; 11 Department of Child and Adolescent Psychiatry, Institute of Psychiatry, Psychology & Neurosciences, King’s College London, London, UK; 12 Psychiatric Neuroscience Group, Department of Basic Medical Sciences, Neuroscience and Sense Organs, University of Bari “Aldo Moro”, Bari, Italy; 13 Psychiatric Imaging Group, MRC London Institute of Medical Sciences, Hammersmith Hospital, Imperial College London, London, UK; 14 Institute of Clinical Sciences (ICS), Faculty of Medicine, Imperial College London, London, UK; 15 Division of Psychology and Mental Health, School of Health Sciences, Faculty of Biology, Medicine, and Health, The University of Manchester, Manchester, UK; 16 Greater Manchester Mental Health NHS Foundation Trust, Addictions Services, Manchester, UK; 17 Department of Psychiatry, Seoul National University Bundang Hospital, Gyeonggi-do, Seoul, Republic of Korea; 18 Department of Psychiatry, College of Medicine, Seoul National University, Seoul, Republic of Korea; 19 Department of Brain & Cognitive Sciences, College of Natural Sciences, Seoul National University, Seoul, Republic of Korea; 20 Division of Neuroscience and Experimental Psychology, School of Biological Sciences, Faculty of Biology, Medicine and Health, University of Manchester, Manchester, UK; 21 Department of Psychiatry, University of Oxford, Oxford, UK; 22 Centre for the Developing Brain, Division of Imaging Sciences & Biomedical Engineering, King’s College London, London, UK

**Keywords:** Neuroscience, Diagnostic markers, Psychiatric disorders

## Abstract

Molecular neuroimaging techniques, like PET and SPECT, offer invaluable insights into the brain’s in-vivo biology and its dysfunction in neuropsychiatric patients. However, the transition of molecular neuroimaging into diagnostics and precision medicine has been limited to a few clinical applications, hindered by issues like practical feasibility, high costs, and high between-subject heterogeneity of neuroimaging measures. In this study, we explore the use of normative modelling (NM) to identify individual patient alterations by describing the physiological variability of molecular functions. NM potentially addresses challenges such as small sample sizes and diverse acquisition protocols typical of molecular neuroimaging studies. We applied NM to two PET radiotracers targeting the dopaminergic system ([^11^C]-(+)-PHNO and [^18^F]FDOPA) to create a reference-cohort model of healthy controls. The models were subsequently utilized on different independent cohorts of patients with psychosis in different disease stages and treatment outcomes. Our results showed that patients with psychosis exhibited a higher degree of extreme deviations (~3-fold increase) than controls, although this pattern was heterogeneous, with minimal overlap of extreme deviations topology (max 20%). We also confirmed that striatal [^18^F]FDOPA signal, when referenced to a normative distribution, can predict treatment response (striatal AUC ROC: 0.77–0.83). In conclusion, our results indicate that normative modelling can be effectively applied to molecular neuroimaging after proper harmonization, enabling insights into disease mechanisms and advancing precision medicine. In addition, the method is valuable in understanding the heterogeneity of patient populations and can contribute to maximising cost efficiency in studies aimed at comparing cases and controls.

## Introduction

Schizophrenia (SCZ) is a severe mental health condition characterized by psychotic symptoms such as a loss of contact with reality, delusions, and abnormal perceptions and behavior. SCZ has been the subject of extensive neuroimaging research to unravel its underlying neurobiological mechanisms. Consistent findings from case-control structural Magnetic Resonance Imaging (MRI) studies have systematically shown brain structure abnormalities, such as reduced grey matter volume and compromised white matter integrity [1]. Similarly, functional MRI (fMRI) studies have identified abnormal connectivity and atypical brain activation patterns during cognitive tasks, suggesting dysfunction in cortical and subcortical regions associated with attention, memory, and emotion regulation [2].

Molecular neuroimaging, such as Positron Emission Tomography (PET), has also provided valuable insights into the molecular alterations associated with SCZ, highlighting the involvement of multiple neuroreceptor systems [3], metabolism [4] and neuroinflammation [5]. The dopamine hypothesis posits dysregulation within the dopaminergic system as a key factor in the development of SCZ and other psychotic disorders [6, 7]. PET studies have consistently shown elevated dopamine synthesis capacity in specific brain regions, particularly the striatum, in individuals with SCZ [8]. Specifically, this hyperactivity of the striatal dopaminergic system is believed to contribute to positive symptoms such as hallucinations and delusions [9–11]. [^18^F]FDOPA PET studies have also provided evidence of altered dopamine-glutamate interactions, highlighting the intricate relationship between these neurotransmitter systems [12]. Interestingly, alterations in dopamine synthesis capacity have also been reported in individuals first-episode psychosis patients (FEP) [13] and individuals at high risk for psychosis [14], highlighting that alterations in dopamine function characterize SCZ at different stages of the disorder.

While offering unique insights into the brain mechanisms underlying SCZ, most neuroimaging literature has primarily focused on identifying single unifying pathophysiological processes shared across patients to identify biomarkers. This has been traditionally addressed using cross-sectional studies that statistically compare group averages, often treating individual differences as noise. However, brain alterations in brain structure and function often overlap between disorders [15]. In addition, psychotic symptoms are not only specific to SCZ as they can also be present in other psychiatric disorders, including bipolar disorder and severe depression [7]. This heterogeneous picture complicates the search for a biomarker in psychiatry [16].

There is a growing awareness that the conventional “average patient” approach falls short of fully capturing the multifaceted characteristics of complex mental health disorders like SCZ and other disorders with psychotic features [17]. In order to develop imaging-based biomarkers with true clinical utility, interindividual variability cannot be simply dismissed as noise or assumed to be part of measurement variability. This awareness has been fostered, in part, by the parallel tendency of collecting large archives of neuroimaging data, which has triggered a gradual shift in neuroimaging towards employing advanced analytical methods to model subjects’ characteristics [18]. By providing statistical inferences at the individual level with respect to an expected pattern, these methods offer the opportunity to parse heterogeneity across cohorts and identify the unique characteristics of each individual. Furthermore, by focusing on personalized perspectives and acknowledging the significance of interindividual variability, these methods have the potential to support the creation for novel neuroimaging biomarkers, to advance our understanding of the neurobiological basis of psychiatric disorders, and potentially aid in predicting treatment outcomes [19, 20].

One of these advanced modelling methods that has gained widespread traction in neuroimaging research is normative modelling (NM, Box 1). This statistical framework is based on the concept of paediatric growth charts [21], which utilise a series of percentile curves to illustrate the normal distribution of children’s body measurements, such as weight, height, and head circumference, as a function of their age. When applied to neuroimaging data [17, 19, 22], this approach enables the identification of a relationship between quantitative neuroimaging biomarkers, such as regional brain volume or thickness measured with MRI, and relevant factors like specific clinical, demographic, or behavioural measures of interest [17].

The rationale for employing normative modelling in neuroimaging is twofold. Firstly, this approach allows us to use neuroimaging data from healthy individuals to establish the normal range for a specific brain characteristic. By describing a portion of the between-subject variability of the given neuroimaging measure through demographic factors (e.g., age, sex [23, 24], BMI) or abilities (e.g., IQ [25]), normative modelling defines what can be considered typical or within the range of expected variation. Secondly, it allows for the quantification of regional deviations from normality at the individual level. This aspect aims to identify disease-specific patterns of alterations and dissect disease heterogeneity across patients. By comparing an individual’s neuroimaging data to the established normal range, it is possible to pinpoint aberrations that may indicate the presence of certain conditions and contribute significantly to the understanding and characterization of brain disorders [19, 26].

Up to now, normative modelling has found extensive application in the identification of consistent disease-specific patterns of brain structural alterations, as demonstrated in schizophrenia [23, 24, 27], bipolar disorder [23, 24], ADHD [28], and Parkinson’s disease [29], using structural MRI data (i.e., T1-weighted MRI [23] and diffusion-weighted imaging [30]). However, this methodology has yet to be applied to the molecular underpinnings of human brain function. The primary challenge in applying normative modelling to PET/SPECT brain imaging lies in the necessity of pooling large datasets to establish reliable parameters for the normative models. To estimate normative models of MRI-based brain measures, hundreds [23, 28, 31] or even thousands [32] of scans are typically included in the reference cohort. However, these numbers cannot be feasibly achieved in single molecular neuroimaging studies due to the substantial costs of PET and SPECT scans (up to 10 times higher than MRI scans) and ethical issues related to the use of radioactive tracers for research purposes. Nevertheless, recent developments in the molecular neuroimaging community, including a greater willingness to share data [33, 34] and the establishment of international consortia (e.g., ENIGMA [35]), along with the development of effective harmonisation techniques for neuroimaging data [36–39], have paved the way for the use of normative modelling in molecular neuroimaging.

In this work, our primary objective is to demonstrate the feasibility of employing normative modelling for molecular neuroimaging in SCZ and psychotic disorders. Despite the challenges posed by small sample sizes and diverse experimental designs, we hypothesise that normative modelling can be effectively applied to this type of data, providing valuable insights into molecular brain functions in healthy individuals and patients. Furthermore, we hypothesise that normative modelling can serve as a powerful tool to identify both the magnitude and the spatial distribution of molecular alterations at the individual patient level by 1) extending analysis of dopamine alterations in the striatum to the whole brain, 2) identifying common patterns of dopamine dysfunction across multiple and independent datasets of patients with SCZ and psychotic disorders, and 3) linking inter-individual deviation from normalities to clinical symptoms and response to treatment.

This study is hence organised into two main parts. In the first section, we used two datasets [40, 41] of [^11^C]-(+)-PHNO PET imaging data measuring D_2/3_ dopamine receptor density, acquired with two scanners, to compare the effects of two image harmonisation methods on the distribution of the deviation scores of the NM and evaluated which one is the most effective at reducing the scanner effects. We then applied the so-identified optimal harmonisation method to studies of dopamine synthesis capacity using [^18^F]FDOPA PET imaging in healthy controls (HC), acquired with five different scanners, and assessed if the [^11^C]-(+)-PHNO results were generalizable to a different radiotracer and a greater number of scanners.

In the second section, we used the estimated [^18^F]FDOPA PET model to calculate the deviations from normality in four datasets [7, 13, 14, 42–48] acquired in patients, one of which did not include any matched HC. Here, we investigated the presence of shared spatial patterns of deviation in patients and the differences between patients with SCZ and FEP. In addition, we tested the feasibility of using independent datasets of patients with no matched HCs when a big enough reference cohort is already available. Here, we assessed the replicability of the findings from the previous comparisons and investigated the clinical value of extreme deviations by looking at relationships between patient-specific extreme deviations from normality and clinical symptoms. Lastly, since [^18^F]FDOPA PET imaging has been recently proposed as a potential biomarker for treatment stratification in psychotic disorders [42], we built a classifier to assess the value of [^18^F]FDOPA PET NM for predicting treatment response to antipsychotics and compared its performances to the reference standard analytics, to evaluate if these would be comparable or outperforming.

Box 1 Normative modelling theory The general framework to derive normative models from neuroimaging data is explained in detail elsewhere [17, 19, 26]. In brief, it comprises several steps (Fig. B1). Firstly, a reference cohort of healthy controls (HC) is chosen. Next, a specific brain measure, such as a given summary measure at the whole-brain level, region-of-interest (ROI)-based derivatives or voxel-wise brain data from structural MRI scans (e.g., volume and cortical thickness), is selected. Additionally, a set of variables (i.e., predictors) is chosen to explain the brain measure. Statistical models are then constructed for each summary measure, establishing a connection between the neuroimaging data and the selected predictors. The primary outcome of the NM is a measure of deviation, typically expressed as a Z-score. This Z-score indicates the extent to which the specific brain measure deviates from the normative distribution, providing valuable information about its deviation from the reference group. Following the model’s estimation, its ability to link individual covariates to neuroimaging data needs to be further evaluated both in-sample (e.g., using k-fold cross-validation) and out-of-sample (e.g., using an independent cohort of HC) [26]. Once the model is validated, it can be used to estimate the deviations of a target cohort (generally corresponding to patients), which can then be analysed by investigating the magnitude and spatial pattern of the so-called “extreme deviations”. These extreme values represent brain areas deviating more than two standard deviations from the reference mean.

Figure B1 Steps of normative modelling. Main steps for the generation and application of normative modelling to neuroimaging data. In brief, the main steps include (1) the assembly of a reference cohort (usually HC) and the selection of covariates of interest (e.g., demographics or cognitive). (2) Model estimation, at desired level of granularity (i.e., voxel- or ROI-level). (3) Model validation, if possible, out-of-sample, or using cross-validation. (4) Application to target cohort, usually patients, and estimation of deviation scores (Z-scores).
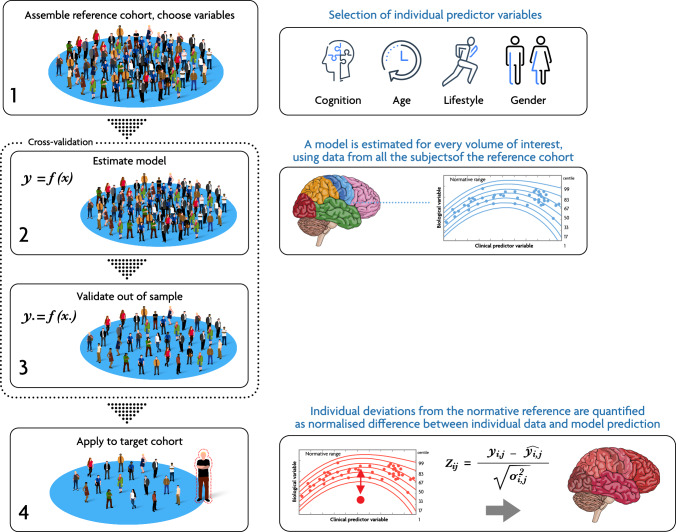

## Methods

### Study datasets

#### [^11^C]-(+)-PHNO Datasets

Data from 77 HC (Table 1) from two previous studies [40, 41], acquired with [^11^C]-(+)-PHNO, were used to assess and select the best image harmonisation method. The scans were acquired with two different scanners (PET/CT, Siemens Hi-Rez Biograph 6, *N* = 54; and PET/MR, Siemens Biograph mMR, *N* = 23) in the same imaging site (Invicro, London).

**Table 1:** Study datasets.

	[^11^C]-(+)-PHNO Datasets										
Group	Age mean (SD) [years]	Sex n (%)	BMI mean (SD) [kg/m^2^]	N. controls	N. patients	N. resp/non-resp (resistant)	Patient Groups	Scanner (scanner type)	Imaging Site	Inclusion Criteria	Clinical Scores
PET/CT	26.52 (7.87)	22F (41%)/ 32M (59%)	23.26 (2.78)	54	n.a.	n.a.	n.a.	Hi-Rez Biograph 6 (PET/CT)	Invicro	HC	n.a.
PET/MR	24.39 (4.90)	12F (52%)/ 11M (48%)	22.47 (2.84)	23	n.a.	n.a.	n.a.	Biograph mMR (PET/MR)	Invicro	HC	n.a.
	**[**^**18**^**F]FDOPA Datasets**										
**Group**	**Age mean (SD) [years]**	**Sex n (%)**	**BMI mean (SD) [kg/m**^**2**^**]**	**N. controls**	**N. patients**	**N. resp/non-resp (or resistant)**	**Patients**	**Scanner (scanner type)**	**Imaging Site**	**Inclusion Criteria**	**Clinical Scores Availability**
FDOPA_01	26.25 (5.18)	32F (36.7%)/ 55M (63.21%)	n.a.	61	25	13/12	First Episode Patients (FEP)	Hi-Rez Biograph 6 (PET/CT)	Invicro	DSM-IV axis I criteria for SCZ (mean Total PANSS [SD]: 72.94 [16.46]), at least one current or previous psychotic episode (duration of illness, median [IQR]: 24 [24] months), medication naïve or free (no medication at scanning with at least 6 months washout for oral medication or 6 months for depot medication	Yes
FDOPA_02	29.00 (8.70)	17F (34.0%)/ 33M (66.0%)	n.a.	26	24	12/12	Chronic	Biograph 40 TruePoint (PET/CT)	South Korea	Chronic illness (duration of illness, mean [SD]: 128 [112.24] months), met the DSM-IV criteria for schizophrenia, had a total score >= 80 in the PANSS Total scale (mean Total PANSS [SD]: 50.0 [9.64]), had received first-line antipsychotic drugs (including risperidone, olanzapine, and paliperidone) or clozapine for at least 12 weeks.	No
FDOPA_03	44.52 (9.97)	21F (58.88%)/ 13M (36.22%)	n.a.	12	24	12/12	Chronic	ECAT HR+962 (PET/CT)	MRC Cyclotron Unit	Chronic illness met the DSM-IV criteria for schizophrenia, paranoid subtype (duration of illness, mean [SD]: 193.8 [367.08] months), received at least two sequential antipsychotic trials (of at least 4 weeks duration) and had a Total PANSS score >= 75. All patients were taking antipsychotic medication at time of scanning other than clozapine.	No
FDOPA_04	30.63 (10.91)	8F (22.22%)/ 28M (77.77%)	n.a.	n.a.	36	20/16	Mixed (FEPs + Chronic)	Hi-Rez Biograph 6 (PET/CT)	Invicro	DSM-V criteria for schizophrenia or schizophreniform disorders, and were not medicated with clozapine in the previous 3 months to scanning	Yes
FDOPA_HC01	29.42 (6.09)	14F (38.89%)/ 22M (61.11%)	n.a.	36	n.a.	n.a.	n.a.	Biograph TruePoint 6 (PET/CT)	Invicro	HC	n.a.
FDOPA_HC02	24.43 (4.65)	1F (14.3%)/6M (85.7%)	n.a.	7	n.a.	n.a.	n.a.	ECAT EXACT 3D (PET/CT)	MRC Cyclotron Unit	HC	n.a.

The table shows datasets of [^11^C]-(+)-PHNO and [^18^F]FDOPA. *n.a. indicates where the data are not available. First Episode Patients (FEP) are patients scanned shortly after the first psychosis episode, while chronic patients are patients who have undergone several rounds of standard-line antipsychotic treatment but have not responded to treatment.

Experimental designs and imaging protocols were consistent between the two studies. Further details on data acquisition, image processing, and data analysis are reported in the original publications [40, 41]. In short, dopamine D2/3 receptor density was measured using non-displaceable binding potential (BP_ND_ [49]) as the parameter of interest. For both scanners, parametric BP_ND_ images were obtained using the same MATLAB-based pipeline with a simplified reference tissue model with cerebellar grey matter used as the reference region. Final maps were all normalised to MNI152 2 mm isotropic standard space before image harmonisation and NM.

#### [^18^F]FDOPA datasets

6-[^18^F]-fluoro-L-DOPA (FDOPA) data from 142 HC and 109 patients with psychotic disorders from previous studies [7, 13, 14, 42–48] were used for estimating the NM of dopamine synthesis capacity and exploration of its alterations in psychosis (Table 1). The datasets consisted of two datasets of HC only (FDOPA_HC01 and FDOPA_HC02), three case-control datasets (FDOPA_01, FDOPA_02, and FDOPA_03) and one dataset of patients only (FDOPA_04).

The FDOPA_01 dataset included FEP patients who were recruited if they had a diagnosis of psychotic disorder according to ICD 10 criteria [50]. FEP patients in the FDOPA_01 dataset were antipsychotic naïve (no current or previous treatment) or free (not taking medication at scanning time with at least 6 months washout for oral medication or 6 months for depot medication) [7]. The FDOPA_02 dataset included patients with a DSM-IV diagnosis of SCZ [45]. Patients in the FDOPA_03 dataset were also patients who met the DSM-IV criteria for SCZ, paranoid subtype. All patients were taking antipsychotic medication at the time of scanning other than clozapine [9]. Lastly, the FDOPA_04 dataset included patients who met the DSM-V criteria for SCZ or schizophreniform disorders [46]. Also, in the FDOPA_04 dataset all patients were taking antipsychotic medication at the time of scanning other than clozapine. Full information on the recruitment criteria and clinical characteristics of the patient population is reported in the original papers [7, 13, 14, 42–48]. For a subset of patients of the FDOPA_01 and FDOPA_04 datasets, symptom severity measures (i.e., Positive and Negative Symptoms Scale – PANSS [51]) were also available.

Data were acquired with five different scanners (Siemens Hi-Rez Biograph 6, Siemens Biograph 40 TruePoint, Siemens TruePoint 6, ECAT HR + 962, and ECAT EXACT 3D) in three different imaging sites (MRC Cyclotron Unit, London; Invicro, London and Bundang Hospital, South Korea). The acquisition protocol was consistent across sites. All FDOPA PET imaging sessions were acquired with a continuous dynamic acquisition (no blood sampling), with scanning beginning with the tracer injection and lasting for ~90 min. All participants received carbidopa (150 mg) and entacapone (400 mg) orally 1 h before imaging to increase the brain tracer uptake and reduce the peripheral formation of radiolabelled metabolite, respectively. The FDOPA tracer (injected dose ranging from 86.4 to 414.4 MBq,) was administered by intravenous bolus injection after the acquisition of a brain CT or MRI for attenuation correction, depending on the scanner availability at each imaging site. PET data reconstruction varied across imaging sites and scanner types, but all included correction for random noise, scatter, and tissue attenuation. Dopamine synthesis capacity indicated by the parameter K_i_^cer^ (sometimes indicated as K_i_, min^−1^) was quantified with the Gjedde-Patlak graphical method using the cerebellum as the reference region. Data were normalised to MNI152 2 mm isotropic standard space using the same in-house pipeline [52], removing data with poor quality and excess of motion. Briefly, the pipeline processes individual FDOPA PET data by assessing total motion and co-registering a tracer-specific template (derived from the average of 10 FDOPA PET scans) to the subject’s motion-corrected PET space. Along with the template, the Martinez [53] and Hammersmith [54] anatomical brain atlases are co-registered to identify regions of interest. Dynamic PET data are quantified at both region and voxel levels, with the latter utilizing a wavelet filter for denoising [55]. Finally, parametric images are normalized to MNI152 standard space using SPM12 (https://www.fil.ion.ucl.ac.uk/spm/) by applying a non-linear transformation field calculated from the participant’s PET summation image.

### Data harmonisation strategies

To harmonise the [^11^C]-(+)-PHNO scans we employed two methods widely used in the literature: Gaussian kernel smoothing [38] and Combat harmonisation [36, 37].

#### Gaussian kernel smoothing (hereafter referred to as “smoothing”)

This method involves applying a 3D convolution filter with a Full Width Half Maximum (FWMH) of 2.35σ, where ${{{\rm{\sigma }}}}$ represents the kernel size. For this step, we used FSL maths [56–58] setting a kernel size of 3 mm, determined through iterative analysis to achieve the best match between the reference and the target data to be harmonised (i.e., PET/CT and PET/MR, respectively).

#### Combat harmonization

Combat is a Bayesian harmonisation method derived from the field of genomics [59], which assumes site-specific scale- and shift-factors to be estimated between different batches (e.g., scanners or sites). Unlike smoothing, Combat allows to estimate and preserve the effect of certain covariates defined a priori on the data variability. In our specific case, we employed the NeuroCombat python library (v.0.2.10+) [36, 37] and preselected the same covariates subsequently used for NM (i.e., age, sex for both tracers and BMI for [^11^C]-(+)-PHNO).

To assess the best harmonisation method, we evaluated the effects of harmonisation on the NM results, particularly focusing on the Z-score distributions and the variance explained by the model (see section ‘Comparison of the harmonisation methods’ below). Once the best method was assessed for HCs this was applied to the patient cohorts directly, as we did not expect a different scanner effect based on the clinical characteristics of the subjects undergoing scanning.

### NM implementation

After data harmonisation, Bayesian Linear Regression (BLR) was used to estimate voxel-wise NMs (i.e., one model per grey matter voxel) of dopamine receptor density and dopamine synthesis capacity from [^11^C]-(+)-PHNO and [^18^F]FDOPA data of the HC samples. BLR is a probabilistic approach to linearly model the relationship between a dependent variable and a set of independent variables. In this case, the selected independent variables were sex, age, and BMI for [^11^C]-(+)-PHNO, and age and sex for [^18^F]FDOPA. Of note, these covariates were used in previous in-house analyses after assessing them to be significant for the specific tracers [52, 60–62].

NMs were estimated using the PCNToolkit (v.1.20.0) Python library, employing default non-informative priors for the parameters and the Powell optimizer for faster convergence of model fitting [19]. Consistently with the PET signal distribution, NM was restricted to the grey matter voxels by thresholding and binarizing a standard probabilistic map of the grey matter at least of 30% probability. Thresholding for GM is not typically applied for FDOPA brain PET imaging [52], but this threshold was used as a trade-off between brain coverage, PET imaging spatial resolution (FWHM between 5 mm and 8 mm) and the computational cost of NM quantification. As a result, only voxels with at least 30% probability of being grey matter were included in the mask, yielding a total of 199,715 voxels (and hence models) for each tracer. Importantly, for the [^11^C]-(+)-PHNO data we estimated three different NMs, i.e., one for the unharmonized data and one for each harmonisation strategy (smoothing and Combat) to identify the strategy that best minimises residual scanner-related differences for PET data. Based on the outcome of this analysis of performance, we then used the identified method to harmonise the [^18^F]FDOPA datasets before the NM step.

The estimated NMs were then used to estimate the voxel-wise maps of deviation from normality for each individual. These maps, expressed in terms of Z-scores, measure the distance of a given data point in relation to the average and standard deviation of the posterior probability, weighted for the subject-specific covariates. These values were estimated using k-fold cross-validation (k = 5) for the HC, or the full [^18^F]FDOPA NM for patients with psychosis. Furthermore, we identified voxels with an extreme deviation from normality (i.e., voxels with PET signal intensity significantly different from the reference distribution). Such extreme deviations were defined using a thresholding approach: Z > 2 for *extreme positive deviations*, Z < −2 for *extreme negative deviations*, and |Z| > 2 for *total extreme deviations*. As the deviation scores follow a normal distribution, it is essential to note that there is an expected percentage of extremely deviating voxels. This consists of 2.5% residual density on each tail of the distribution (i.e., Z > 2 or Z < 2), totalling 5% for the absolute value (i.e., |Z| > 2). Conversely, we anticipate a higher percentage of extremely deviating voxels, either positive or negative, in subjects deviating from normality.

### Identification of the optimal harmonisation strategy based on [^11^C]-(+)-PHNO data

For each harmonisation strategy (no harmonisation, harmonisation with smoothing and harmonisation with COMBAT), we compared the average and standard deviation of the individual Z-score distributions between the two scanners (i.e., PET/CT and PET/MR) to identify any residual differences between datasets. These comparisons were performed using Mann-Whitney U tests, implemented with the rstatix package [63] in R (version 4.2.1). Furthermore, using the brain parcellation defined by the Hammersmith atlas [54], for each harmonisation strategy we estimated the regional percentage of extreme positive and negative deviations across subjects of each dataset (i.e., PET/CT and PET/MR data). We then calculated the regional differences between the two datasets and employed Wilcoxon’s two-sample rank-tests implemented with the rstatix package [63] in R 4.2.1 to identify any significant scanner-related differences.

### Validation of the optimal harmonisation strategy on [^18^F]FDOPA data

We validated the harmonization method identified as optimal for the [^11^C]-(+)-PHNO data on the [^18^F]FDOPA data to see if it would yield consistent results in terms of removing any scanner-related differences across datasets.

First, we harmonised the [^18^F]FDOPA HC data with the optimal harmonisation strategy identified using the [^11^C]-(+)-PHNO data. Then, to include in the analysis only those voxels for which the NMs were able to describe the between-subject variability based on the variables used to estimate the model (i.e., age and sex for the [^18^F]FDOPA data), we discarded the grey matter voxels where NMs were not able to converge to a solution, i.e., those reporting an explained variance (EXPV) lower than 0. Furthermore, since a positive yet very small EXPV does not necessarily mean that the model can meaningfully describe the data, we decided to restrict the subsequent analyses to voxels with EXPV > 3% (i.e., voxels where NM reached statistical significance of p_uncorr_ < 0.05). For completeness, we repeated the analyses on the data, including voxels with EXPV > 0, and using a conservative threshold of EXPV ≥ 10%, corresponding to the optimal point from the L-curve distribution of EXPV.

Finally, to validate the optimal harmonisation strategy on [^18^F]FDOPA HC data, we calculated the mean and standard deviation of the individual distributions of HC Z-score deviations and compared them by non-parametric Kruskal-Wallis H test (rstatix [63] library in R 4.2.1) as the distributions were not normal.

### NM of [^18^F]FDOPA PET in patients with Schizophrenia and FEP

After calculating the z-score deviations of the three clinical cohorts (i.e., FDOPA_01, FDOPA_02, and FDOPA_03) using the NMs estimated from the HC data, we grouped the Z-score distributions for HC and patients separately and compared them. We also calculated the Risk-Ratios (RR) [64] by counting the number of voxels whose Z-scores showed extreme deviations from normality, using the epitools library in R 4.2.1. In this case, the RR indicates the likelihood of a new individual belonging to the clinical cohort as compared to the group of HCs.

We then tested the hypothesis that patients would show a greater overlap of extreme deviations than HC, indicating spatial consistency in the manifestation of the disorder, by investigating the spatial patterns of extreme deviations in HC and patients. This was done by counting, for each voxel and group, the number of subjects showing an extreme deviation in that voxel. This was assessed separately on the positive and negative extreme deviations and on the total extreme deviations. This spatial analysis was performed to identify the possible presence of specific brain areas consistently involved in the manifestation of the disease.

We then analysed the differences between HC and patients in terms of the magnitude of deviation from normality by performing both a voxel-wise analysis on the Z-score maps (both whole-brain grey matter and masked by extreme deviations), and on summary measures of deviations. The voxel-wise analysis allows spatial localization of the significant differences between the two groups, whereas summary measures determine whether it is possible to collapse meaningful information into a few scores per subject, which might be useful for clinical applications. The four summary scores analysed were the mean Z-score, corresponding to the mean Z-score across voxels for each subject, and three measures of extreme deviations from normality, estimated by counting the percentage of voxels with Z-score > 2 (i.e., positive deviations), Z-score <−2 (i.e., negative deviations) or |Z-score| > 2 (i.e., total deviations). Since the clinical datasets included subjects with different diagnoses (i.e., FEP and schizophrenia), we ran the non-parametric Schreier-Ray-Hare Tests to investigate the main effects of the cohort and their interaction. On the other hand, the voxel-wise tests were performed using FSL *randomise* [65], with 5000 permutations and considering a cluster significant if p_FWE_ < 0.05 [66, 67], corrected for multiple comparisons using the threshold-free cluster enhancement [66] (TFCE) option. For the significant contrasts, we extracted the mean z-score values from the significant clusters and performed post-hoc tests using the rstatix [63] library in R 4.2.1. For the tests on the summary metrics, we used the rcompanion and rstatix [63] library in R 4.2.1.

Lastly, we investigated which biological pathways were driving the spatial pattern of deviations identified in the voxel-wise analysis. This was done by running imaging-transcriptomics with the imaging-transcriptomics toolbox [68–70] on the F-stat map reporting the main effect of the group, and by running GSEA on molecular function, as defined by the GO Molecular Function [71, 72].

### Extending normative model validity to an independent patient cohort

To demonstrate the value of NM analysis when using a clinical cohort without its own group of matched HCs and test the replicability of the results from the previous analyses, we applied the [^18^F]FDOPA PET NM on an independent dataset of patients with SCZ (FDOPA_04). Of note, although this clinical cohort was independent, the scanner effect was modelled in the estimated NM since this dataset was acquired at the same site and using the same scanner as the FDOPA_01 dataset. We estimated the four summary measures (mean Z-score and positive, negative, and total extreme deviations) in this cohort and compared them with the summary measures of the HC of the other datasets (FDOPA 01, 02 and 03). For this analysis, we performed non parametric Mann-Whitney U test using the rstatix [63] library (R 4.2.1).

### Relationships between deviation scores and clinical symptoms

In datasets where clinical data were available, specifically FDOPA_01 and FDOPA_04, we tested the hypothesis that NM deviation scores would be associated with symptom severity, as measured using PANSS scores [51] using voxel-wise comparisons and summary measures, on each of the two datasets independently. Voxel-wise covariance analysis was conducted utilizing FSL *randomise* [57, 58, 65] (independent variables: PANSS scores; dependent variable: Z-score), while Spearman correlations between whole brain summary measures and PANSS scores were computed using rstatix [63] (R 4.2.1). For the cohort of patients without matched controls (FDOPA_04), we additionally performed a correlation analysis between the individual PANSS scores and the average Z-scores of the statistically significant clusters, identified from the voxel-wise cross-sectional analysis (Spearman correlation, rstatix [63] library, R 4.2.1).

### Prediction of treatment response in patients

To determine whether the predictive power of FDOPA summary measures outperformed reference analysis (i.e., striatal K_i_), in predicting antipsychotic treatment response for each clinical cohort we constructed ROC curves for all summary measures of the deviation scores (average Z-score, positive, negative, and total extreme deviations) across the whole brain and striatum and ROC curves for the original striatal K_i_ measures. ROC curves were compared using the DeLong test. All analyses were performed using the pROC library [73] R 4.2.1.

Response to treatment was defined differently for each FDOPA dataset. In FDOPA_01, response was characterized by a reduction of more than 50% in PANSS total symptom severity from baseline to follow-up, with this improvement sustained for at least 6 months. Conversely, non-response was identified by a reduction of less than 50% in symptom severity, with no evidence of improvement over the same period [13, 42]. For the FDOPA_02 dataset, treatment response was determined by the Remission in Schizophrenia Working Group criteria for remission [9, 42, 74], whereas non-response was based on the modified Kane criteria for treatment resistance in schizophrenia [9, 42, 75]. In the original FDOPA_03 study, responders were those patients who had received first-line antipsychotic drugs (such as risperidone, olanzapine, or paliperidone) for a minimum of two weeks, without a history of clozapine administration. Non-responders, on the other hand, were those who had not responded to at least two first-line antipsychotic drugs, as documented through chart review [45]. Lastly, in FDOPA_04 responders were defined as patients who had treated with only 1 medication since onset of the disease, if any medication was changed in those patients it was due to adverse events rather than non-response. Additionally, they had a CGI-SCH severity score <4, a PANSS total score <60 and a compliance rating scale score >3. Non-responders were defined as patients who had received treatment with at least 2 antipsychotic medication for >4 weeks each (in doses above the minimum therapeutic doses), had a CGI-SCH score >30, a total PANSS score >70 and a compliance rating scale score >3 [46].

## Results

### Estimation of NM for [^11^C]-(+)-PHNO PET and assessment of the optimal harmonisation strategy

**Fig. 1:**
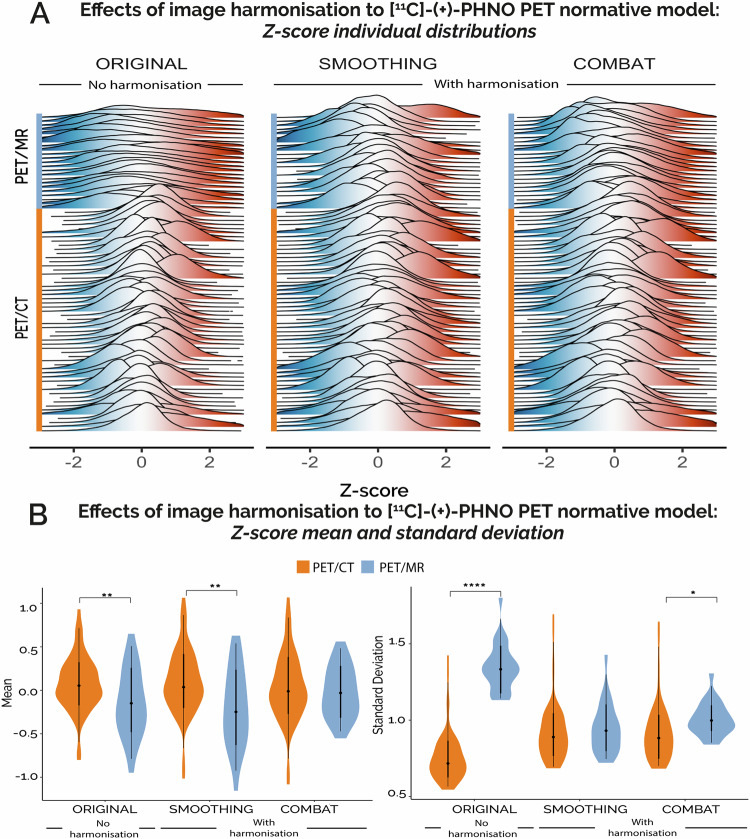
Harmonisation results of [^11^C]-(+)-PHNO PET normative modelling. **A** Single-subject, whole-brain distributions of Z-scores estimated in the data before harmonisation (original data, on the left) and after spatial smoothing (in the centre) and Combat (on the right) harmonisation methods. This dataset combines scans of healthy controls acquired with either PET/CT (N = 54) or PET/MR (*N* = 23) scanners. **B** Welch two-sample t-tests comparing the first statistical moments (mean and standard deviation of the single-subject Z-score distributions between scanner types for each harmonisation modality (no harmonisation, harmonisation with spatial smoothing and harmonisation with Combat). Orange violin plots refer to PET/CT scans while blue violin plots refer to PET/MR scans. Asterisks indicates significance. *indicates *p* < 0.05, **indicates *p* < 0.01 and ****indicates *p* < <0.001.

As shown in Fig. 1A, the Z-score distributions of the non-harmonised [^11^C]-(+)-PHNO PET data present two distinct scanner-related patterns, which are attenuated after harmonisation with both harmonisation strategies. When comparing the averages of the distributions between scanner types, we found statistically significant differences between the PET/CT and PET/MR scanners both in the unharmonized data (r = 0.29 [0.05, 0.5], Z = 2.52, *p* < 0.05) and in the data harmonised with smoothing (r = 0.32 [0.08, 0.55], Z = 2.78, *p* < 0.01), while there were no scanner-related differences in the data harmonised with Combat (r = 0.07 [0.004, 0.29], Z = 0.67, *p* = n.s.). In terms of standard deviation, we found significant differences between scanner types in the non-harmonised data (r = 0.75 [0.64, 0.83], Z = −6.61, *p* < 0.001), in the data harmonised with Combat (r = 0.34 [0.15, 0.51], Z = −3.03, *p* < 0.01), while no differences between scanner types were found in the data harmonised with smoothing (r = 0.08 [0.004, 0.29], *p* = n.s.).

When analysing between-scanner differences at the regional level (Supplementary Fig. 1), all harmonisation methods showed significant differences in the number of extreme deviations between scanner types (*p* < 0.01), except Combat for the negative extreme deviations. Overall, Combat returned the smallest differences, hence better harmonisation performances, for both positive extreme deviations (No harmonisation: Δ_MR-CT_ = 4.30 ± 2.21%; Smoothing: Δ_MR-CT_ = −1.03 ± 1.39%; Combat: Δ_MR-CT_ = 0.44 ± 1.04) and negative extreme deviations (Original: Δ_MR-CT_ = 6.02 ± 2.30%; Smoothing: Δ_MR-CT_ = 2.06 ± 2.10%; Combat: Δ_MR-CT_ = 0.06 ± 0.97%) (Supplementary Fig. 1).

### Validation of the optimal harmonisation strategy on [^18^F]FDOPA data

After harmonising [^18^F]FDOPA data with Combat, we estimated the voxel specific NMs, which explained up to 15% of the data variability although they do not converge to a solution for approximately 56% of the grey matter masked implemented (i.e., EXPV < 0; Supplementary Fig. 2). All subsequent results will refer to NM data thresholded with the 3% variance mask, although the same analyses were repeated for the other masks (Supplementary Tables 1–12).

No clear scanner effects were apparent from the individual distributions of Z-scores in HC (Supplementary Fig. 3). Similar to [^11^C]-(+)-PHNO PET NM, for FDOPA a small scanner-related effect size (η^2^ = −0.022, 95% CI: [−0.02, 0.06]) was found in terms of mean z-score distribution (χ^2^ = 0.972, *p* = n.s), while a moderate effect size (η^2^ = 0.094, 95% CI: [0.02, 0.25]) was found for the standard deviation (χ^2^ = 16.9, *p* < 0.05). These numbers confirmed that the chosen data harmonisation strategy was effective for the FDOPA datasets.

### NM application to [^18^F]FDOPA PET in SCZ and FEP

The group pooled distributions (i.e., all subjects, all voxels) of HCs and patients showed small differences in the mean (|Δ_μ_^HC-PAT^| = 0.14) and a greater difference in terms of the standard deviation (|Δ_σ_^HC-PAT^| = 0.28), consistently higher for patients in both measures. The number of extremely deviating voxels (in percentage to the total voxels in the analysis mask), above the level of chance, shows significant increased RR for patients with all extreme deviation scores (RR_pos_ = 2.19, 95% CI: [1.97, 3.45], *p* < 0.001; RR_neg_ = 1.92, 95% CI: [1.60, 3.00], *p* < 0.001; and RR_tot_ =2.60, 95% CI: [1.36, 2.70], *p* < 0.001 for positive-, negative-, and total-extreme deviations respectively) (Supplementary Fig. 4).

**Fig. 2:**
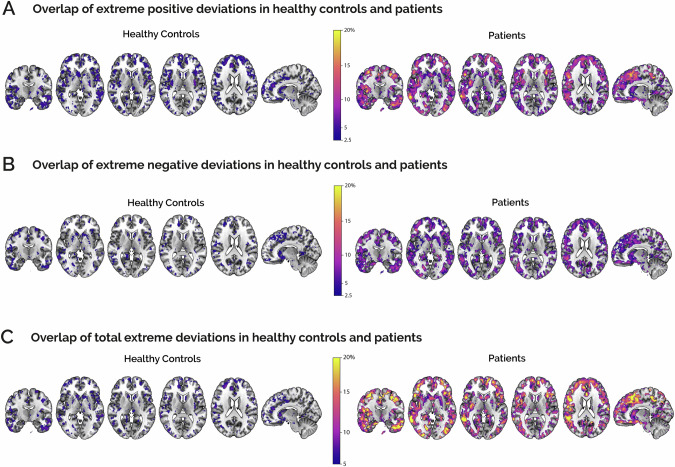
Overlap maps of extreme deviations in HC and patients. Each map reports for each voxel the percentage of subjects with co-localised positive (**A**), negative (**B**) and total (**C**) extreme deviations. Controls (left) and psychosis patients (right) are reported separately. The figure is set at a threshold above the level of chance (2.5% for positive and negative deviations and 5% for total deviations). All overlap maps are superimposed on a standard MNI152 structural template.

We hypothesised that the HC group would exhibit a relatively low percentage of individuals with extreme deviations, while conversely, patients would demonstrate a higher overlap in the percentage of individuals with extreme deviations from normality in specific brain regions. Indeed, HCs reported very low percentages of individuals with overlapping extreme deviations, which aligns with the NM hypothesis for the reference cohort (Fig. 2). On the contrary, patients exhibited distinct spatial patterns of extreme deviations, primarily in the cerebral cortex (Fig. 2). These deviations were prominently present in the precentral and frontal gyri, with the major discrepancy being reported for extreme positive deviations and total extreme deviations (Fig. 2A, C) However, it is important to emphasise that this pattern of extreme deviations also showed substantial sample heterogeneity, as the highest percentage of patients with comparable extreme deviations did not exceed 20%.

**Fig. 3:**
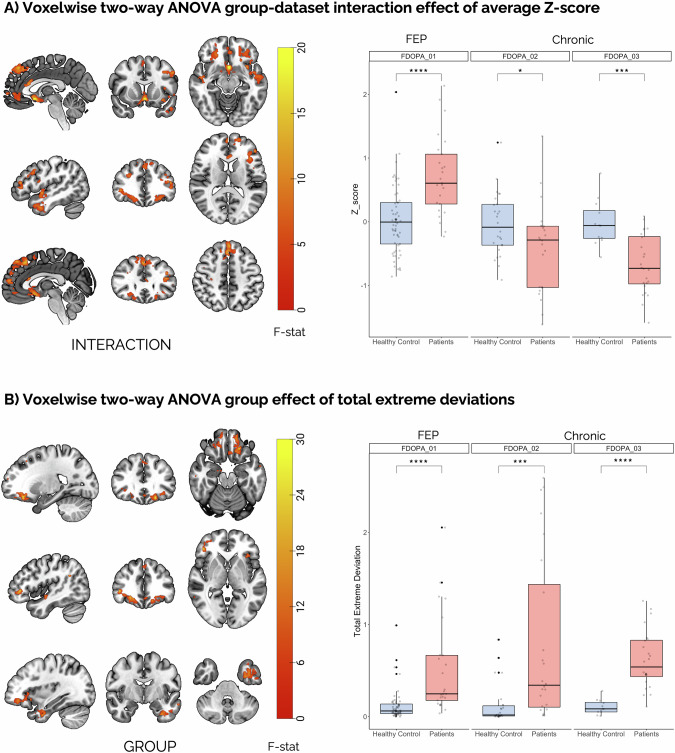
Voxel-wise ANOVA analysis of deviation scores, expressed as mean Z-score and total extreme deviations, for the [^18^F]FDOPA Normative Model. (A) Mean Z-score significant clusters (left) and post-hoc analyses, grouped by dataset, (right) between healthy controls and patients. (B) Total extreme deviations significant clusters (left) and post-hoc analyses, grouped by dataset, (right) between healthy controls and patients. *indicates *p* < 0.05, ***indicates *p* < 0.001 and ****indicates *p* < <0.001.

The voxel-wise analysis performed on the Z-score maps (two-way ANOVA following test for Gaussianity) revealed significant interactions between group and dataset (F_peak_=21.71, p_peak_ < 0.001 FWE corrected, Fig. 3A). This interaction spanned the temporal lobe, the medial- and superior-orbitofrontal, and the frontal lobe. The post-hoc tests to evaluate between-group differences within each dataset highlighted greater positive deviations in the FEP as compared to the HC (FDOPA_01, t = 4.92, *p* < <0.001) and lower negative deviations in SCZ patients as compared to HC in both FDOPA_02 (t = 2.22, *p* < 0.05) and FDOPA_03 (t = 4.62, *p* < 0.001).

We also found a group effect by repeating the analysis on the Z-score maps thresholded by |Z| > 2 (F_peak_=34.36, *p* < 0.001 FWE corrected) (Fig. 3B), exhibiting an increase in patients. This effect was localised in the left frontal orbital cortex, frontal pole, and inferior temporal cortex. Of note, only clusters including more than 50 voxels are reported.

**Fig. 4:**
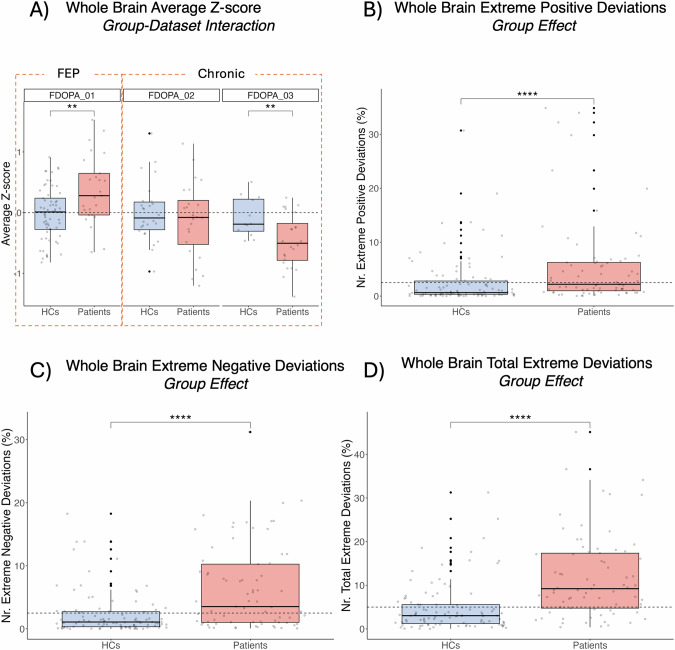
ANOVA analysis of individual deviation scores, expressed as mean Z-score and positive, negative, and total extreme deviations, for the [^18^F]FDOPA Normative Model. (A) Mean Z-score differences between healthy controls and patients grouped by dataset. (B) Difference of extreme positive deviations between healthy controls and patients grouped by dataset (C) Difference of extreme negative deviations between healthy controls and patients grouped by dataset. (D) Difference of total extreme deviations between group. Patients are composed of a mixture of first episode (FDOPA_01) and chronic psychosis (FDOPA_02, FDOPA_03), depending on the dataset. Asterisks indicates significance, * indicates *p* < 0.05, ** indicates *p* < 0.01 and **** indicates *p* < <0.001.

Schreier-Ray-Hare tests conducted on the summary measures of the mean z-score and extreme positive, negative, and total deviations revealed a significant interaction between group and dataset for the mean Z-score (H = 9.66, *p* < 0.01; Fig. 4A). Post hoc analyses showed a moderate increase, in average Z-score in FEP (χ^2^ = 8.44, *p* < 0.01, η^2^ = 0.089, 95% CI: [−0.002, 0.24]), and a moderate decrease in SCZ patients (FDOPA_03, χ^2^ = 7.03, *p* < 0.01, η^2^ = 0.177, 95% CI: [0.0001, 0.44]) compared to HCs. Similarly, the FEP group was associated with a moderate increase in extreme positive deviations compared to HCs (χ^2^ = 19.8, *p* < <0.001, η^2^ = 0.223, 95% CI: [0.09, 0.38]). The positive, negative, and total extreme deviations did not exhibit a significant interaction between the group and dataset, although it did indicate a significant effect of the group (positive: H = 18.06, *p* < <0.001; negative: H = 9.51, *p* < 0.01; total: H = 26.52, *p* < <0.001, Fig. 4B–D), showing an increase in patients compared to HC. Post-hoc analyses with the Kruskal-Wallis H test indicated a moderate increase between HC and both patient groups in extreme positive (χ^2^ = 18.1, *p* < <0.001, η^2^ = 0.101, 95% CI: [0.03, 0.21]) and negative (χ^2^ = 20.9, *p* < <0.001, η^2^ = 0.117, 95% CI: [0.03, 0.22]) deviations, whereas total extreme deviations exhibited a large difference (χ^2^ = 36.4, *p* < <0.001, η^2^ = 0.208, 95% CI: [0.10, 0.33]). Furthermore, the Schreier-Ray-Hare tests indicated a significant effect of the dataset in both extreme positive (H = 6.16, *p* < 0.05), negative (H = 23.72, *p* < <0.001) and total deviations (H = 11.17, *p* < 0.01).

The imaging transcriptomic analysis conducted on the F-stat maps resulting from the significant group effect, regardless of the duration of psychosis, on the total extreme deviations, tested with two-way ANOVA assessing and revealed several notable enriched gene sets. These included “structural constituent of ribosome” (NES = 2.49, p_FDR_ < 0.05), “neurotransmitter receptor activity” (NES = 2.18, p_FDR_ < 0.05) and “glutamate receptor activity” (NES = 2.04, p_FDR_ < 0.05), as determined by the GO molecular function [71, 72].

### Extending normative model validity to an independent patient cohort

When visually compared with the HC of the independent datasets in terms of the percentage of individuals with extreme deviations (positive, negative, and total) within the group, the pattern of extreme deviations of the FDOPA_04 dataset is consistent with that of the datasets of patients in FDOPA_01, FDOPA_02, and FDOPA_03 (Supplementary Figs. 5–7).

In terms of differences in the summary measures (mean z-score and positive, negative, and total extreme deviations) between FDOPA_04 patients and HC, we found significant increases in patients in terms of mean Z-score (U = 3853, *p* < <0.001), extreme positive deviation (U = 4308, *p* < <0.001) and total extreme deviations (U = 4476.5, *p* < <0.001). No significant differences were found in terms of negative extreme deviations (U = 2719, *p* = n.s.).

### Correlation between imaging-based deviations from normality and clinical symptoms

We tested the hypothesis that there would be an association between the significant clusters identified from the voxel-wise analysis and PANSS scores in both the FDOPA_01 (FEP, included in the voxel-wise analysis) and FDOPA_04 (SCZ, independent dataset) datasets. There were no significant correlations in the FDOPA_01 dataset. In the FDOPA_04 dataset, clinical scores were significantly correlated with the mean Z-scores (PANSS positive: ρ = 0.36, *p* < 0.05; PANSS negative: ρ = 0.38, *p* < 0.05; PANSS general: ρ = 0.41, *p* < 0.05; PANSS total: ρ = 0.43, *p* < 0.01; Table 2) and total extreme deviations (PANSS positive: ρ = 0.36, *p* < 0.05; PANSS negative: ρ = 0.34, *p* < 0.05; PANSS general: ρ = 0.42, *p* < 0.05; PANSS total: ρ = 0.44, *p* < 0.01;Table 2). Three individual clusters in FDOPA_04 (with n_voxels_ ≥ 50) survived correction for multiple comparisons, showing significant correlations with total PANSS scores (Cluster 1: ρ = 0.41, *p* < 0.05; Cluster 2: ρ = 0.36, *p* < 0.05; Cluster 3: ρ = 0.43, *p* < 0.05; Table 3).

**Table 2:** Correlations between significant voxel-wise clusters and PANSS scores.

	PANSS Positive		PANSS Negative		PANSS General		PANSS Total	
	FDOPA_01	FDOPA_04	FDOPA_01	FDOPA_04	FDOPA_01	FDOPA_04	FDOPA_01	FDOPA_04
**Average Z-score**	0.38	0.36*	0.38	0.38*	−0.06	0.41*	0.36	0.43**
**Extreme total deviations**	0.32	0.36*	0.31	0.34*	−0.09	0.42*	0.25	0.44**

The table shows Spearman correlation’s rho and relative p-value for significant clusters resulting from voxel-wise two-way ANOVA (average Z-score, and |Z| > 2). Correlations are separated for FDOPA_01 (FEP patients, N = 25) and FDOPA_04 (chronic and FEP patients, N = 36).

* and bold indicates p < 0.05.

** and bold indicates p < 0.01.

**Table 3:** Correlation analysis of PANSS total scores and individual voxel-wise clusters.

Spearman correlation with PANSS Total scores			
	Correlation	Cluster size	Cluster Location
**Cluster 1**	0.41*	505	Frontal Orbital Cortex, Frontal Pole
**Cluster 2**	0.36*	421	Frontal Orbital Cortex, Frontal Pole, Inferior Frontal Gyrus
**Cluster 3**	0.43*	50	Middle Temporal Gyrus

Table shows Spearman correlation’s rho and relative p-value for significant clusters between total extreme deviations and PANSS Total Scores in FDOPA_04 (chronic patients), resulting from voxel-wise two-way ANOVA (|Z| > 2). Cluster locations were obtained using FSL atlasquery from the Harvard-Oxford Cortical Atlas.

* and bold indicates p < 0.05.

Correlation of the summary deviations and PANSS scores (Table 4) revealed a significant correlation between total extreme deviations and PANSS negative in FEP patients for FDOPA_01 dataset (ρ = 0.47, *p* < 0.05). Similarly, the analysis of FDOPA_04 reveals significant correlations between extreme positive deviations and PANSS general scores (ρ = 0.33, *p* < 0.05) and between total extreme deviations and all PANSS scores (PANSS positive: ρ = 0.33, *p* < 0.05; PANSS negative: ρ = 0.39, *p* < 0.01; PANSS general: ρ = 0.44, *p* < 0.01; PANSS total: ρ = 0.46, *p* < 0.01).

**Table 4:** Correlation analysis of PANSS scores and summary measures.

	PANSS Positive		PANSS Negative		PANSS General		PANSS Total	
	FDOPA_01	FDOPA_04	FDOPA_01	FDOPA_04	FDOPA_01	FDOPA_04	FDOPA_01	FDOPA_04
**Average Z-score**	0.28	0.25	0.30	0.16	−0.11	0.24	0.17	0.22
**Extreme positive deviation**	0.33	0.29	0.36	0.17	−0.12	0.33*	0.24	0.31
**Extreme negative deviations**	−0.07	−0.12	0.14	0.06	0.19	−0.01	0.11	0.03
**Extreme total deviations**	0.36	0.33*	0.47*	0.39**	−0.01	0.44**	0.30	0.46**

Table shows Spearman correlation’s rho and relative p-value for whole brain summary measures (average Z-score, Z > 2, Z < -2, |Z| > 2). Correlations are separated for FDOPA_01 (FEP patients, N = 25) and FDOPA_04 (chronic and FEP patients, N = 36).

* and bold indicates p < 0.05.

** and bold indicates p < 0.01.

### Treatment response in patients

**Fig. 5:**
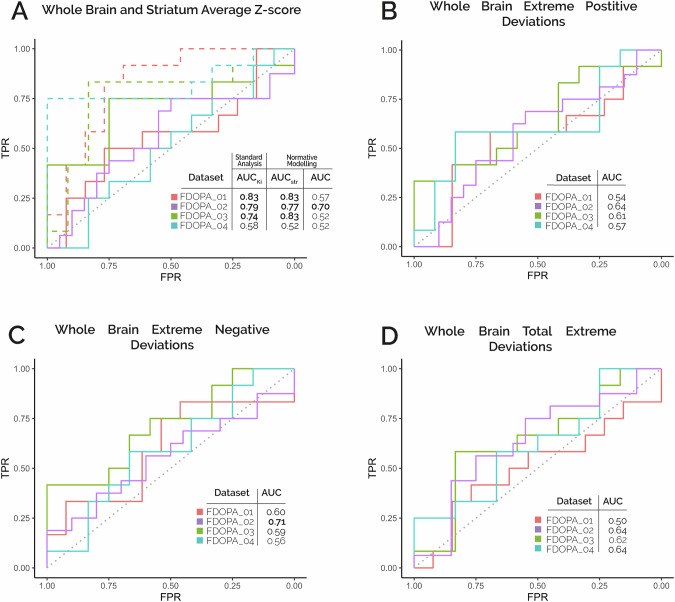
[^18^F]FDOPA Normative Model Treatment Response. ROC curves for the classification of treatment response (Responders vs Non-Responders/Resistant) using whole brain (solid lines) or striatal (dashed lines) individual average Z-score **A**, whole brain positive extreme deviations **B**, negative extreme deviations **C** and total extreme deviations **D** for the different clinical datasets. Dashed diagonal indicates the level of chance in all plots. All scores refer to [^18^F]FDOPA patient data after Combat harmonization. Bold numbers in figure tables show significant (*p* < 0.05) correlations.

The analysis of the mean striatal z-score measures to evaluate patients’ response to standard antipsychotics replicated the performance of the reference analysis (i.e., based on striatal dopamine synthesis capacity proxied by K_i_^cer^), and no significant differences were found between the two methods (Fig. 5A). Here we obtained acceptable classification performances for FDOPA_02 (AUC_str_=0.77, AUC_Ki_ = 0.79) [76], and excellent classification performances for FDOPA_01 (AUC_str_=0.83, AUC_Ki_ = 0.83) and FDOPA_03 (AUC_str_=0.83, AUC_Ki_ = 0.74) [76]. However, when extending the analysis to the mean z-score measures estimated at the whole brain, we did not reach statistically significant classifications except for FDOPA_02 (AUC = 0.70, Fig. 5A). Similarly, the classification of treatment response measured with whole-brain deviation scores pointed at acceptable [76] performances only for FDOPA_02 in terms of negative extreme deviations (AUC = 0.72, Fig. 5C). All the other classification attempts did not reach statistical significance.

## Discussion

This study applied normative modelling (NM) in PET imaging of the dopamine system in patients with SCZ and FEP using several existing datasets. Two dopaminergic radiotracers ([^11^C]-(+)-PHNO for D_2/3_ receptor and [^18^F]FDOPA for dopamine synthesis) were used to construct normative models from reference healthy controls and applied to patients with psychosis. We demonstrate that NM can explain dopamine function variability arising from demographic factors and show that patients display different patterns of extreme deviations. Indeed, [^18^F]FDOPA PET showed high patient heterogeneity, particularly in extra-striatal brain areas. In the striatum, deviation scores could classify treatment response, although with similar performance to standard kinetic modelling analysis. Overall, NM provides novel quantitative insights into the brain dopamine system in healthy individuals and SCZ, taking advantage of existing normative data from multiple neuroimaging studies.

### Considerations on NM applied to molecular neuroimaging

In our study, we first confirmed the necessity of data harmonisation to compile multi-centre, multi-scanner PET datasets with the required sample size for constructing a normative model of healthy cohorts. Our results indicated that Combat outperformed the smoothing method to remove scanner and site effects. This finding aligns with the neuroimaging literature, where model-based harmonization methods are the preferred choices in PET [77] and other modalities [36, 37]. It is worth noting that Combat assumes linearity in the scanner’s effect, which may not hold true for tomographs with different geometries, axial fields of view, and gamma detectors. Recently, non-linear versions of Combat methods (e.g., Combat-GAM [78]) have been introduced, but they would require further testing with PET tracers exhibiting different kinetic properties. Regardless of the type of harmonization statistics used, our proposed approach relies on the existence of a group of healthy controls (HC) for scanner calibration. This results in a non-generalizable model without HC data. One solution proposed in other neuroimaging modalities [31, 32, 79] could be to incorporate the scanner directly into the normative model as a random effect using hierarchical modeling. While appealing for modelling flexibility and the absence of data pre-harmonisation, this methodology is burdened by extreme computational times (reported to be up to ~24 h per model [31]), making it unfeasible for our application (i.e., ~130 K voxel-wise models). Moreover, with a limited sample size (as in this paper), hierarchical modeling could lead to biased results in the parameter distributions [80].

Our normative model was able to explain a substantial amount of dopamine function variability (~30% in D_2/3_ receptor density, as measured by PHNO, and ~15% in dopamine synthesis capacity, as measured by FDOPA) using simple demographic factors from the population. This level of variance is lower than that reported in other modalities (40% for sMRI [39], 29% for diffusion MRI [81], and 2–23% for molecular enriched networks [82]), which may relate to measurement of brain functional processes rather than structure. To allow generalizability, the covariates we selected were limited to general demographic factors (age and sex for [^18^F]FDOPA; age, sex, and BMI for [^11^C]-(+)-PHNO), which were not specific to psychiatric disorders. Although the addition of more diagnosis-specific covariates and/or additional experimental factors (e.g., genetic traits) may have improved the normative model’s performance, the use of ancillary measures (e.g., genetic tests or clinical assessments) might limit its potential clinical utility and generalisability due to the necessity of having a PET scan and additional testing.

Moreover, our study explored the feasibility of investigating dopaminergic alterations of [^18^F]FDOPA without acquiring a matched set of healthy controls. With the analysis of the patient-only cohort (i.e., FDOPA_04) and its comparison to the whole normative reference (i.e., all HCs), we obtained similar results as those in the other clinical datasets which had matched HCs in the normative reference, including the spatial pattern of the extreme deviations. This finding is particularly relevant, as routine clinical applications typically do not acquire matched controls for patients. The NM approach circumvents this issue using a normative reference, accounting for covariates for comparison. This framework, along with automatic standardised frameworks for kinetic modelling of [^18^F]FDOPA [52], represents a significant step forward in establishing PET parametric imaging as a quantitative companion tool for the clinical management of psychosis patients. It mirrors, in some respects, the routine use of DAT SPECT imaging for quantifying dopamine transporter in Parkinson’s disease and differentiates it from essential tremor [83, 84]. Furthermore, integrating a normative reference population could significantly reduce, if not eliminate, the need to acquire matched healthy controls (HC) in future research studies. This reduction could be achieved by replacing a portion of the HC subjects with model-based predictions, thereby reducing costs and experimental time and, most importantly, minimizing participants’ unnecessary exposure to radioactivity. In this context, the availability of a large dataset of healthy control PET scans could serve as a valuable resource for any PET radiotracer and target, supporting clinical trials across various brain disorders.

### Implications for understanding pathophysiology in schizophrenia and other psychotic disorders

Our results reveal novel pathophysiological insights for the understanding of SCZ and other psychotic disorders. Firstly, we observed that patients with SCZ and FEP exhibit a higher percentage of extreme deviations than HCs. This is in line with the result of cross-sectional studies that consistently reported elevated DSC in patients with schizophrenia as compared to healthy controls in the striatum. However, the application of the NM framework allowed us to expand current evidence by showing that patients with schizophrenia and FEP also show an elevated risk ratio (RR) for both positive and negative extreme deviations in patients compared to controls. While further investigations with radiotracers with higher specific binding for extrastriatal regions are needed to refine our models, these results add to previous evidence showing alterations in dopamine function in schizophrenia also outside the striatum [85].

In addition, our individual modelling approach allowed us to identify differences in dopamine function between individuals at different disease stages. Indeed, we observed that the pattern of positive and negative extreme deviations varied between FEP and patients with SCZ. For example, FEP patients show a higher number of positive extreme deviations than healthy controls and chronic SCZ patients, while the latter show a higher number of negative extreme deviations than controls and FEP patients. This pattern is also evident in the average distribution scores and is confirmed by significant voxel-wise analyses (average Z-score and total extreme deviations). When testing differences in deviation scores in striatal regions, we did not identify differences between FEP and SCZ (Supplementary Fig. 9), and the whole patient group showed a higher number of positive deviations than controls. FDOPA signal does not seem to be affected by antipsychotic treatment, at least in the short term [47]. Still, the effect of long-term exposure antipsychotic exposure on striatal dopamine synthesis capacity is unknown. Our result, showing higher positive deviations in patients than controls regardless of the disease stage seems to suggest that elevated dopamine synthesis capacity in the striatum persists in chronically medicated patients, but further testing is needed to confirm this hypothesis. The neurobiological interpretation of higher cortical negative deviations in chronic patients as compared to FEP is more challenging. Our cortical results might align with previous findings with other imaging modalities, indicating a progressive decline in cortical metabolism and function [4], paralleled by structural remodelling, such as lower cortical volume and thinner cortices in chronic SCZ patients compared to FEP [86]. Evaluation of deviation in FDOPA signal from reference-cohort in longitudinal datasets and the integration of other imaging modalities might confirm this hypothesis. With the data available to our study, however, we cannot establish whether this differential pattern between FEP and chronic patients is attributed to variances in disease duration or is linked to medication effects. In fact, at the current state we were unable to test for causality of previous medication and disease duration on the differential pattern exhibited by the different cohorts. It is worth noting that each patient’s experience with the disease is unique, and ideally, they should not be grouped with other patients for analytical purposes but rather considered as individual “clusters“ [87]. Nevertheless, by employing NM, it would be feasible (provided the requisite data is available) to analysed individual-level deviation with consideration to their clinical and disease history. Achieving this will require collecting and harmonizing all relevant non-imaging data, with a particular focus on clinical data pertinent to the intended use case. Without individualized response variables, it would remain impossible to draw meaningful inferences about the practical utility of normative modelling methods applied to neuroimaging.

Another interesting finding from our analyses is the substantial heterogeneity among patients, even though there is a consistent effect in all patients. This heterogeneity is particularly noticeable in analysis of the overlap of extreme deviations, where at most, 20% of patients co-localize in the same voxels. The co-localization of extreme deviations is more pronounced in patients compared to healthy controls (with a maximum expected overlap of 5%), indicating a common alteration of [^18^F]FDOPA PET signal in patients. These areas of extreme deviations are consistent with previous findings on NM applied to structural MRI, showing a maximum degree of overlap of 5–10% [23, 24]. Future studies should explore of interlinks between structural and molecular changes at the individual level. Interestingly, [^18^F]FDOPA PET co-localizations of extreme deviations in psychosis occur in extra-striatal brain areas, contrary to the focus of most [^18^F]FDOPA studies on striatal areas [7, 13, 14, 42–48, 52]. Studies that have examined the cortical signal of [^18^F]FDOPA suggest elevations in dopamine synthesis capacity in cortical areas (e.g., posterior cingulate [88], medial prefrontal cortex [89]) or correlations between dopamine synthesis capacity in cortical areas and clinical symptom severity (e.g., a positive correlation between PANSS positive scores and K_i_ in the right temporal cortex [90]). In addition, the deviation scores of FDOPA_04, especially total deviations, showed significant correlations with PANSS symptoms, both as whole-brain summary scores and significant cluster measures. Interestingly, by analysing individual clusters within the cluster signal, we found that the clusters correlating with total PANSS symptoms were in the frontal and temporal gyri. However, no significant correlations were observed in the smaller FDOPA_01 dataset, which may relate to lower statistical power. These findings raise questions about alterations in aromatic L-amino acid decarboxylase (AADC) and whether patients have an unresponsive feedback mechanism of tyrosine hydroxylase, which may not down-regulate as in normal populations [88]. Early animal studies suggest that the [^18^F]FDOPA signal combines metabolic activity related to dopamine synthesis, storage, and metabolism [91]. Our imaging-transcriptomics analysis partially confirms these findings pointing towards an association between genetic pathways related to metabolism and [^18^F]FDOPA deviations measured in psychosis. All these findings suggest that cortical signal of [^18^F]FDOPA might be associated with increased cellular transport and/or protein metabolism, supporting the application of this biomarker in neuro-oncology [92]. Further studies are required to fully characterise the nature of the [^18^F]FDOPA signal in the human cortex, establishing the contributions of dopamine function (e.g., neuron density and changes in dopaminergic-mediated neuronal firing), metabolism and cellular transport to the overall signal. Lastly, there are genetic variants that are well-known to influence the dopamine system (e.g., 22q11.2 deletion/duplication [43] and COMT Val^158^Met polymorphism [93]) or that have been associated with the [^18^F]FDOPA PET signal (e.g., AS3MT/BORCS7 genetic variant [94]). These factors could drive functional differences observed between patients and HCs and increase the inter-individual explained variance if included in the NM. Further analyses in a larger sample with full genetic information are warranted.

### Implications for biomarkers of treatment response

To date, treatment of SCZ and other psychotic disorders is mainly empirical, and no clinically useful biomarkers to predict response to antipsychotic treatment are available. Therefore, we also evaluated the potential of using NM-derived scores for classifying treatment response in patients and compared these findings to previously published results [42]. Interestingly, when focusing on striatum NM, we replicated the treatment prediction performances of the standard analysis method [42], while poor classification performances were obtained when extending NM to the whole brain. This outcome aligns with expectations, as the molecular target of action of both antipsychotic medications is striatal dopamine D_2/3_ receptors [95, 96]. In fact, the striatal average Z-scores (as defined by the Hammersmith atlas and where the model converges) were highly correlated with K_i_ estimates in all patient datasets (0.93 < R < 0.97, *p* < 0.001, Supplementary Fig. 8), providing thus an alternative view of the same information. In line with this, the distributions of extreme positive and total extreme deviations in the striatal region revealed group differences, indicating an elevation in the dopamine signal (Supplementary Fig. 9). Additionally, the better performances in the striatal regions compared to whole brain measures might be reflective of the superior sensitivity of the FDOPA radiotracer in this region, as compared to the rest of the brain [8–10, 38–47]. Moreover, it is important to highlight the fact that the definition of treatment response varied across individual studies, partly explaining the difference in classification performances between datasets.

### Limitations

This work is subject to several limitations. Firstly, the modelling process is influenced by the harmonization method and may not account for non-linearities within the system, either in harmonization or modelling. Despite our efforts to harmonize data from different clinical sites, residual differences still exist in the datasets when assessed with statistics of the second order or above (e.g., skewness, kurtosis). Secondly, applying NM at the voxel level can result in underperformance of the model due to high noise and poor signal. Consequently, there are a considerable number of voxels where the model fails to converge. This issue could potentially be addressed by adjusting the Markov Chain Monte Carlo (MCMC) settings or by incorporating a more extensive set of covariates to describe the neuroimaging target variable. MCMC is used in Bayesian modelling, and NM, to approximate the posterior distribution of parameters by generating samples from the distribution through iterative random sampling [97]. While NM mapping provides a whole brain topological description of patient deviation, region-based modelling remains a valid compromise between spatial resolution and method performance. In our study, however, we did not investigate regional models in order to avoid that the model be linked to a particular brain parcellation or regions associated with any particular disorder or to avoid the masking of potential sub-regional effects (e.g., dorsal vs ventral striatum) which might not be captured with inappropriate brain parcellations. Thirdly, the deviation scores might be sensitive to motion [98], however when assessing for our data there were no significant correlations in HCs nor in patients (Supplementary Fig. 10). Fourthly, the heterogeneity of the clinical sample used in the analysis and the presence of missing clinical information for some individuals in the cohort are likely to have affected statistical power. Even if our dataset can be considered a large one for a molecular neuroimaging study, it is indeed modest compared to normative modelling MRI studies. It is difficult to provide a retrospective power calculation of our sample as there are many factors influencing it (e.g. the choice of Bayesian statistics for NM, the uncertainty of its prior, or the strength of associations between covariates and PET measures). Based on our simulations (data not shown), for [^18^F]FDOPA PET we recommend a reference 50 scans and in case of multiple scans/sites, at least 10 normative subjects each.

Lastly, our result could be influenced by the choice of EXPV threshold used for masking (3%) and statistical tests. For consistency with literature [22], we repeated the analysis with parametric tests and obtained similar results for the summary measures (Supplementary Tables 1–12). The only difference between was found in the significance of the interaction (group and dataset) of extreme positive and negative deviations. This discrepancy can be explained by the limitations of the Schreier-Hare-Ray test [99]. Moreover, it is important to stress that, while Gaussianity is one of the assumptions of ANOVA testing, it has been shown that the infringement of this has virtually no effect in type-I errors for the test [100]. Another limitation is that our datasets included patients with SCZ and FEP, which are distinct nosological entities. While a high proportion of FEP subsequently develop SCZ [101], FEP cohorts also included diagnoses other than schizophrenia, such as affective psychosis (e.g. bipolar disorder or major depression with psychotic features) or unspecified psychosis). This might, at least in part, explain the heterogeneity in dopamine function in the FEP cohort described here. Future longitudinal studies with a higher refinement of inclusion criteria in the FEP cohort (e.g. schizophreniform disorder) could improve our understanding of progression of dopamine alterations in schizophrenia.

## Conclusions

In conclusion, the NM framework can be successfully applied to molecular neuroimaging (i.e., PET and SPECT) after proper harmonisation of scanner effects. Moreover, with the NM model, we can assess a differential pattern of deviations in patients with SCZ and FEP and compare a patient-only cohort to the normative reference to gain mechanistic insights and advance toward a quantitative and biological understanding of psychosis. Additionally, we were able to replicate the findings of traditional cross-sectional studies and performances with standard analytical approaches. While the focus of this work was on the presynaptic dopaminergic system (as measured by [^18^F]FDOPA) and on the post-synaptic dopaminergic system (as measured by [^11^C]-(+)-PHNO) our results encourage the adoption of this analytical framework to any molecular target measured by PET or SPECT neuroimaging and in other neuropsychiatric disorders.

## Supplementary information

Supplemental_material_PET_NM

## Data Availability

The data that support the findings of this study are available from The NeurOimaging DatabasE (NODE) repository (https://maudsleybrc.nihr.ac.uk/research/precision-psychiatr y/neuroimaging/neuroimaging-database-node/) but restrictions apply to the availability of these data, which were used under license for the current study, and so are not publicly available. Data are however available from the authors upon reasonable request and with permission by the data controller institutions, by contacting the support team (node.information@kcl.ac.uk) or the author Dr. Mattia Veronese (mattia.veronese@kcl.ac.uk).
