# Correction: Investigating dopaminergic abnormalities in schizophrenia and first-episode psychosis with normative modelling and multisite molecular neuroimaging

**DOI:** 10.1038/s41380-025-02987-1

**Published:** 2025-03-31

**Authors:** Alessio Giacomel, Daniel Martins, Giovanna Nordio, Rubaida Easmin, Oliver Howes, Pierluigi Selvaggi, Steven C. R. Williams, Federico Turkheimer, Marius De Groot, Ottavia Dipasquale, Mattia Veronese, the FDOPA PET imaging working group, Ilinca Angelescu, Micheal Bloomfield, Ilaria Bonoldi, Faith Borgan, Tarik Dahoun, Enrico D’Ambrosio, Arsime Demjaha, Jecek Donocik, Alice Egerton, Sameer Jauhar, Stephen Kaar, Euitae Kim, Seoyoung Kim, James Maccabe, Julian Matthews, Robert McCutcheon, Philip McGuire, Chiara Nosarti, Matthew Nour, Maria Rogdaki, Grazia Rutigliano, Peter S. Talbot, Luke Vano

**Affiliations:** 1 Centre for Neuroimaging Sciences, Institute of Psychology, Psychiatry and Neuroscience (IoPPN), King’s College London, London, UK; 2 Division of Adult Psychiatry, Department of Psychiatry, Geneva University Hospitals, Rue Gabrielle Perret-Gentil 4, 1205 Geneva, Switzerland; 3 Department of Psychosis Studies, Institute of Psychology, Psychiatry and Neuroscience (IoPPN), King’s College London, London, UK; 4 MRC Laboratory of Medical Science, Imperial College London, London, UK; 5 South London and Maudsley NHS Foundation Trust, London, UK; 6 Department of Translational Biomedicine and Neuroscience, University of Bari “Aldo Moro”, Bari, Italy; 7 GSK R&D, Clinical Pharmacology and Experimental Medicine, Clinical Imaging, Stevenage, UK; 8 Department of Information Engineering, University of Padova, Padova, Italy; 9 Department of Psychosis Studies, Institute of Psychiatry, Psychology & Neuroscience, King’s College London, London, UK; 10 Division of Psychiatry, Faculty of Brain Sciences, University College of London, London, UK; 11 Department of Child and Adolescent Psychiatry, Institute of Psychiatry, Psychology & Neurosciences, King’s College London, London, UK; 12 Psychiatric Neuroscience Group, Department of Basic Medical Sciences, Neuroscience and Sense Organs, University of Bari “Aldo Moro”, Bari, Italy; 13 Psychiatric Imaging Group, MRC London Institute of Medical Sciences, Hammersmith Hospital, Imperial College London, London, UK; 14 Institute of Clinical Sciences (ICS), Faculty of Medicine, Imperial College London, London, UK; 15 Division of Psychology and Mental Health, School of Health Sciences, Faculty of Biology, Medicine, and Health, The University of Manchester, Manchester, UK; 16 Greater Manchester Mental Health NHS Foundation Trust, Addictions Services, Manchester, UK; 17 Department of Psychiatry, Seoul National University Bundang Hospital, Gyeonggi-do, Seoul, Republic of Korea; 18 Department of Psychiatry, College of Medicine, Seoul National University, Seoul, Republic of Korea; 19 Department of Brain & Cognitive Sciences, College of Natural Sciences, Seoul National University, Seoul, Republic of Korea; 20 Division of Neuroscience and Experimental Psychology, School of Biological Sciences, Faculty of Biology, Medicine and Health, University of Manchester, Manchester, UK; 21 Department of Psychiatry, University of Oxford, Oxford, UK; 22 Centre for the Developing Brain, Division of Imaging Sciences & Biomedical Engineering, King’s College London, London, UK

**Keywords:** Neuroscience, Diagnostic markers, Psychiatric disorders

Correction to: *Molecular Psychiatry* 10.1038/s41380-025-02938-w, published online 28 February 2025

In this article, the order in which the authors appeared in the author list was incorrectly given as Ilinca Angelescu^9^, Micheal Bloomfield^10^, Ilaria Bonoldi^9^, Faith Borgan^9^, Tarik Dahoun^11^, Enrico D’Ambrosio^9,12^, Arsime Demjaha^9^, Jecek Donocik^5^, Alice Egerton^9^, Stephen Kaar^9,13,14,15,16^, Euitae Kim^17,18,19^, Seoyoung Kim^17^, James Maccabe^9^, Julian Matthews^20^, Robert McCutcheon^9,21^, Philip McGuire^21^, Chiara Nosarti^11,22^, Matthew Nour^21^, Maria Rogdaki^9^, Grazia Rutigliano^9,13^, Peter S. Talbot^20^ and Luke Vano^5,9,13^ where it should have been Ilinca Angelescu^9^, Micheal Bloomfield^10^, Ilaria Bonoldi^9^, Faith Borgan^9^, Tarik Dahoun^11^, Enrico D'Ambrosio^9,12^, Arsime Demjaha^9^, Jecek Donocik^5^, Alice Egerton^9^, Sameer Jauhar^9,13,14^, Stephen Kaar^9,13,14,15,16^, Euitae Kim^17,18,19^, Seoyoung Kim^17^, James Maccabe^9^, Julian Matthews^20^, Robert McCutcheon^9,21^, Philip McGuire^21^, Chiara Nosarti^11,22^, Matthew Nour^21^, Maria Rogdaki^9^, Grazia Rutigliano^9,13^, Peter S. Talbot^20^ and Luke Vano^5,9,13^.

The original article has been corrected.

